# Research on the Audit Prediction Model of “Special Bonds + PPP” Project based on Machine Learning

**DOI:** 10.1155/2022/4174473

**Published:** 2022-08-11

**Authors:** Qianru Fang

**Affiliations:** Wenzhou Polytechnic, Wenzhou Higher Education Park, Wenzhou 325000, Zhejiang, China

## Abstract

This paper aims at the whole-process tracking audit problem of “special bonds + PPP” mode (hereinafter referred to as “special bonds + PPP”) in public infrastructure construction projects and establishes an audit evaluation prediction model based on the theory and method of machine learning. Firstly, based on expert interviews and the actual working process of “special bonds + PPP,” the comprehensive evaluation index system of the whole process tracking audit is established. Secondly, innovate audit technology methods and apply machine learning theories and methods such as support vector machine, back propagation neural network, multinomial logistic regression, and random forest to the whole tracking audit of “special bonds + PPP.” Finally, the real case evaluation sample data are selected, and the four established models, that is, SVM, BP, Multinom, and RF, are trained and predicted. After comparative analysis, the RF model with the highest accuracy is selected as the evaluation prediction model.

## 1. Introduction

With the continuous enhancement of China's comprehensive national strength, the demand for public infrastructure construction projects is surging. The PPP mode has become the main starting point of national infrastructure construction, but its financing difficulties are increasingly prominent. Such as local government special bonds have the advantages of the short issuance review period and low financing cost, and the “special bonds + PPP” mode of public infrastructure construction projects (hereinafter referred to as “special bonds + PPP”) financing mode emerges. It has become the main mode of government project investment and financing. At present, how to ensure the legality of the operation of “special bonds + PPP” and guarantee the project benefits is of great importance. A set of consistent whole-process tracking audit index system is needed, and an innovative evaluation method is needed to establish an audit prediction model to achieve effective supervision and audit prediction.

In domestic and foreign literature research and project practice, we divided scholars' literature on PPP project tracking audit and special debt audit into the following three categories:

  The first category is the research on key indicators of PPP project audit. Cheung [1] used the expert scoring method for multiparty evaluation and identifies effective risk, output benefits, project management ability, and technological innovation ability as key indicators of PPP project audit. Lu [2] drew lessons from the management mechanism of India and proposed that China should strengthen audit in key elements, work process, audit focus, risk assessment, and other aspects of PPP project. Jiang and Xu [3] proposed to start with the whole project cycle, focusing on the selection of optimal solutions, project progress, project investment, risk management and control, completion quality, project operation, and other key points to implement whole-process audit coverage, based on the comparison of the advantages and disadvantages of the VFM evaluation system both domestic and abroad. Li [4]suggested that a PPP project audit should focus on construction project quality, internal control, investment estimation, risk management, and other aspects. Wang and Zhang [5] believed that the key points of project audit are project screening, legal compliance of the implementation process, policy implementation, and investment performance. Ye et al. [6] proposed that the partnership value, economy, efficiency, and effectiveness are the main contents of the audit of the PPP project. Based on the perspective of “dual identity” of government, Wang and Yan [7] believed that bonds risk, public interest, financial bearing capacity, and value for money evaluation report et al. are the key points of audit. Liu [8] studied the risk-oriented audit theory and put forward investment contract, policy implementation, social investment, construction management, and economic benefits as audit priorities. Liu [9] proposed the construction of an audit indicator system from the perspective of the project process, taking project stages of identification, preparation, procurement, implementation, and handover as dimensions.
  The second category is the study of PPP project audit evaluation methods. Tang [10] summarized the current problems of PPP project audit efficiency and advocated using big data to carry out PPP project audit so as to avoid the shortage of sample reviews. Xiong [11] proposed that the application of cloud computing, big data, and other technology development means, as well as the application of audit tools, provide support for the full coverage of PPP project audit. Drawing on the PPP case of the British metro, Liang [12] proposed to use modern information technology execution and information transmission to build a trinity mechanism of entrustment to ensure full audit coverage. Liu et al. [13] established the VFM evaluation model and analyzed the PSC value of the comprehensive pipe gallery project and the LCC value in its PPP mode through actual cases. Xiang and Song [14] analyzed the interaction between risk factors of PPP projects and established a system dynamics model to strengthen the monitoring of financing risks. Zhao et al. [15] established a comprehensive performance rating model based on the matter-element extension evaluation method and evaluated PPP project performance index values as the matter-element to be evaluated. Liu et al. [16] used the BCC model and Malmquist index to evaluate the public governance performance of the PPP model in 31 provinces and cities from 2017 to 2019. Qin et al. [17] determined the index weight based on decision laboratory analysis (DEMATEL) and network analytic hierarchy process (ANP) and established the dynamic evaluation model of financing risk by using the extended approximate ideal solution (TOPSIS). Chang et al. [18] established the extension matter-element model of financing risk and used the correlation degree between the evaluation indexes and grades of the correlation degree function to evaluate the financing risk grade of the PPP project.
  The third category is the key research of special bonds audit. Xiaoquan [19] believed that strengthening audit intensity in the whole life cycle of special bonds is conducive to reducing the risk of special bonds. Wen et al. [20] proposed to strengthen audit of special bonds projects from three aspects: progress of bonds fund use, disclosure of bonds information, and investment and use of bonds funds, so as to prevent risks and improve benefits. Chai [21] believed that in the operation of government special bonds projects, the mode of bond issuance, the duration of bond issuance, and the mode of principal and interest repayment should be strengthened. Xu and Yang [22] analyzed the risk points of special bonds and summarized the rights and responsibilities of bonds issuing subjects, bonds size and structure, project selection, capital use efficiency, and sources of bonds repayment funds et al. as audit priorities. Cai [23] believed that the key point of special bonds supervision is whether the bidding, capital management, and construction management et al. of construction project and material procurement are standardized.

The above-given research focuses on the major links and key indicators of PPP projects and special debt audit, and PPP project evaluation methods. To a certain extent, they have ensured that the value for money, project progress control and risk control of single models such as PPP mode and special bonds mode meet the expected objectives. As an innovative mode with Chinese characteristics, special bonds and PPP combined mode is an innovative mode to solve the financing difficulties of the PPP mode. Currently, there are few studies that combine the application of artificial intelligence algorithms such as machine learning. In terms of audit evaluation and prediction, there is a lack of overall index systems and intelligent evaluation predicting tools for the audit supervision of “special bonds + PPP.”

Based on the research results of scholars, we propose to establish an appropriate audit evaluation and prediction model based on the theory and method of machine learning to carry out the whole-process tracking audit evaluation and prediction of “special bonds + PPP.” First of all, based on expert interviews and combined with the declaration and actual working process of “special bonds + PPP,” the key points of audit are identified, and the comprehensive evaluation index system of the whole process tracking audit is established, striving to cover all indicators. Secondly, innovate audit technology methods and apply machine learning theories and methods such as support vector machine, back propagation neural network, multinomial logistic regression, and random forest to the whole tracking audit of “special bonds + PPP.” Finally, design the experts evaluation questionnaire, select the real case, obtain original sample data, process the input, and output data in a quantitative way, and the four established models, that is, SVM, BP, Multinom, and RF, are trained and predicted. After comparative analysis, the RF model with the highest accuracy is selected as the evaluation prediction model. Meanwhile, this model is easy to operate and has a high level of versatility, which offers a theoretical and intelligent technical support for the “special bonds + PPP” audit activity coverage.

## 2. Construction of Whole-Process Tracking Audit Indicator System of “Special Bonds + PPP”

Based on the previous research and with expert interviews, this paper analyzes the audit focus of PPP project and special bonds, respectively, combines with the actual declaration and work process of “special bonds + PPP,” and based on the principle of full life cycle tracking audit coverage. The original evaluation indicators are extracted from the three dimensions of early project approval decision, midterm supervision and management, and later handover evaluation as the whole process tracking audit of “special bonds + PPP.” Then, select the national registered supervision engineers with practical work experience, senior engineers of the audit bureau, engineers of design institutes, college teachers, and scientific researchers with relevant knowledge as the survey objects. Through the “Sojump” app, the original audit evaluation index questionnaire is distributed to the respondents and analyzed, so as to build a whole process tracking audit evaluation index system including 3 primary indicators(as A), 23 secondary indicators (as B), and 68 tertiary indicators (as C), as shown in Table 1.

## 3. Data Source and Application

### 3.1. Sample Data Source and Pretreatment

This paper selects the real project of “special bonds + PPP,” which was selected by the ministry of finance, the project of the Zouping freight railway special line in Binzhou City, Shandong Province, as a case. Take the tertiary indicators C_1_–C_68_ and the overall evaluation of the project in Table 1 “special debt + PPP” whole process followup audit evaluation index system,” a total of 69 evaluation indicators, as the content of the questionnaire. Each evaluation index is set as excellent, good, medium, and poor in the four options. 50 questionnaires were sent to the registered supervision engineers of relevant engineering project management companies and 39 valid questionnaires were recovered. By analyzing the survey results of the overall evaluation item, 92.31% of the supervision engineers think the project is excellent or good, indicating that the project runs well. Therefore, the case data is used in the research of the next audit prediction model. Since the 69 index data collected in the questionnaire are descriptive data, they are analyzed and processed quantitatively according to the evaluation level from high to low. For C_1_–C_68_ indicators, 4 represents excellent, 3 represents good, 2 represents medium and 1 represents poor, the overall evaluation of the project is still expressed in four grades: excellent, good, medium, and poor. Sample data are excerpted in Table 2.

### 3.2. Application of Model Index Data

The valid sample data *X*={*X*_1_,…, *Xn*}, *Y*={*Y*_1_,…, *Yn*}, *n*=1,…, 39 of numbered 1–39 processed by the method described in “2 sample data sources and preprocessing” in this paper is adopted. C_1_–C_68_ index data in each sample *X*_*n*_=(*x*_1_,…, *x*_*i*_), *i*=1,2,…, 68 were taken as input data and overall evaluation data *Y*_*n*_, (*n*=1,2,…, 39) as output data. Using machine learning theories and methods such as support vector machine, back propagation neural network, multinomial logistic regression, and random forest, four models SVM, BP, Multinom, and RF are established, respectively.

The application process of valid sample data is as follows: (1) data are randomly classified. 50% of sample data are randomly selected from 39 data samples as the model training set (as TRS), and the other 50% of sample data as the model testing set (as TES). The testing set is independent of the training set and does not participate in training at all and is only used for the final evaluation of the model effect. (2) *K*-fold cross validation is adopted. In order to make better use of the limited sample data, increase the sample size and ensure the stability of generalization error, so as to obtain the ideal model, *K*-fold cross validation is needed. The training set is divided into *K* folds, of which *K* − 1 folds are used as *K* − 1 training sets(as TRS1∼TRSK−1). By determining the fitting curve parameters, the classification model is established, and *K* different models are obtained, which are recorded as *M*, *M*={*M*_1_,…, *M*_*K*_}. The *k*-th fold is used as the validation set (as VAS) to evaluate the effect of the model *M* and select the best hyper parameter to optimize the model. The above-given process was verified *K* times without a repeated sampling cycle to obtain the final required model. In this paper, according to the sample size, *K* = 5 is set to carry out a 5-fold cross-validation, training, and tuning of the model. (3) Final evaluation of model effectiveness. The four optimal models of SVM, BP, Multinom, and RF obtained after training are tested for their prediction accuracy through the testing set, and the prediction results are compared with the overall evaluation of the project of the samples in the testing set to select the most appropriate model.

## 4. Analysis of Modeling Ideas of Machine Learning-Related Models

### 4.1. Support Vector Machine Algorithm Model (as SVM)

Support Vector Machine algorithm is a supervised learning and training classifier algorithm, whose goal is to accurately classify data by solving a geometrically spaced maximum hyperplane. The advantage of SVM is that it is based on the principle of structural risk minimization, which is suitable for the small sample situation existing in this paper and has good robustness. It helps us to grasp the key samples and delete redundant samples, thus overcoming the problems of “dimension disaster” and “overlearning” to a large extent.

The modeling idea is as follows:(1) given a training set *S*={*X*_*n*_, *Y*_*n*_}, *X*_*n*′_ ∈ *X* ∈ *R*^*i*^, *n*′=(1.2,…, 20), *n*′ is the number of training set samples, _*X*_ as input space, each point *X*_*n*′_ in the input space is composed of *i* attribute features as support vectors, and *Y*_*n*′_ ∈ *Y* ∈ {−1, 1} is the target function to output data. In this paper, each *X* of the independent variable *X*_*n*′_ in the audit prediction has 68 attribute features, that is, the C_1_–C_68_ index data in each sample is recorded as *X*_*n*′_={*x*_*n*′1_, *x*_*n*′2_,…, *x*_*n*′68_} as the input vector. The dependent variable *Y*_*n*′_ is the overall evaluation of the project as the regression output value. (2) When the training set data is linear, on the basis of maximizing the hard interval, the relaxation variable *ξ* ≥ 1 is introduced to maximize the soft interval, and the linear classifier model is learned and trained. (3) When the data of the training set is nonlinear, the inner product kernel function *K*(*x*_*i*_, *x*_*j*_) is introduced, and the input vector *X*_*n*′_ is mapped to a high-dimensional linear feature space, so that *Y*_*n*′_=*F*(*x*_*n*′1_, *x*_*n*′2_,…, *x*_*n*′68_) trains the classifier in the high-dimensional feature space, and the decision function is obtained. (4) The relevant parameters of the separation hyperplane *w*(*x*) with the largest geometric interval were determined and the SVM model was obtained. (5) The above steps, combined with the *K*-fold cross-validation method, were used to train the model with the training set and tune the model with the testing set to determine the optimal parameters and obtain the optimal SVM model.

### 4.2. Back Propagation Neural Network Algorithm Model (as BP)

Back Propagation neural network is a multilayer feedforward neural network, which is the most widely used neural network model and a powerful data modeling tool. In essence, the algorithm takes the global sum of squares error of the network as the objective function and calculates the minimum value by using the gradient method. The advantage of BP lies in its ability to process the nonlinear transformed information with minimum input error. The ability of self-learning, self-adaptation, and generalization is good, and the fault tolerance rate is high [24].

**Figure 1:**
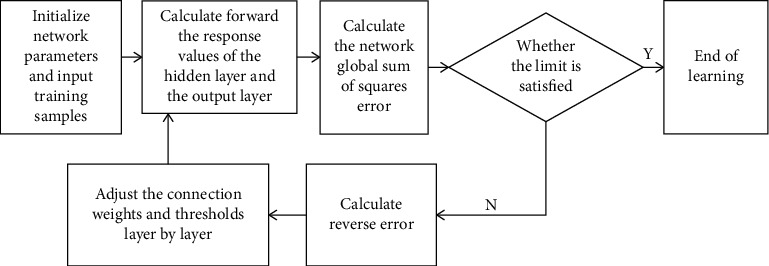
Modeling schematic diagram of back propagation neural network.

The modeling idea is as follows (see Figure 1): (1) Back Propagation neural network structure is established. This paper adopts a single-layer feedforward neural network, which is composed of three neural network models: input layer *X*_*n*_, *X*_*n*_=(*x*_1_,…, *x*_68_), middle layer (1 hidden layer), and output layer *Y*_*n*_. Neuron nodes of adjacent layers are fully connected by connection weight. (2) Back Propagation neural network training is divided into signal forward propagation and error reverse feedback, and the training set is input into the network with a predetermined limiting value. After single hidden layer processing, when the global sum of squares error of the network meets the limiting value, that is, the output value is consistent with the output of the expected response, the training learning ends. If the global sum of squares error does not meet the limit value, that is, the output value is inconsistent with the expected response output, then the corrected error value is calculated and the connection weights and thresholds are modified layer by layer according to the back propagation network. (3) With the *K*-fold cross-validation method, the training set and verification set are trained and verified repeatedly until the global square sum error meets the limit value, so as to obtain the minimum error value and obtain the optimal BP model.

### 4.3. Multinomial Logistic Regression Algorithm Model (as Multinom)

Multinomial Logistic Regression algorithm is a machine learning classification algorithm based on probabilistic nonlinear regression. The goal is to estimate the probability value through the logistic function, It is used to measure the relationship between dependent variables and multiple independent variables (characteristics). The advantage of multinomial is that through the convenient observation sample probability score, it can fundamentally solve the problem of how to do if the dependent variable is noncontinuous, the calculation cost is not high, and it is easy to understand and implement.

The modeling idea is as follows: (1) analyze the characteristics of valid samples *S*={*X*_*n*_, *Y*_*n*_}, *n*=1,…, 39 in this paper. The independent variable in each sample has 68 characteristic attributes, that is, C_1_–C_68_ index data in each sample, indicating that the number of events in this group is 68. The dependent variable *Y* is unique, that is, the overall evaluation of the project is one of the four grades of excellent, good, medium, and poor. Each grade is orderly, which belongs to the situation of multiclassification and order of response variables. Therefore, the ordered cumulative probability logistic regression model is established according to the characteristics of the samples. (2) Combining the *K*-fold cross-validation method with the maximum probability estimation method, the training set, and verification set are used to train and test continuously, and the optimal parameters are found to maximize the joint probability, and the optimal Multinom model is obtained.

### 4.4. Random Forest (RF) Algorithm Model

Random Forest is a supervised learning integration algorithm based on if-then-else rules. It generates many decision trees by randomly extracting variables and sample data and then summarizes the results of decision trees to become a random forest algorithm model. The advantage of RF is that it avoids the limitations of a single model. The accuracy of the integrated model is better than that of most single algorithms. The introduction of the randomness of feature subset and feature quantity makes RF not easy to fall into overfitting, fast training speed, and strong universality.

**Figure 2:**
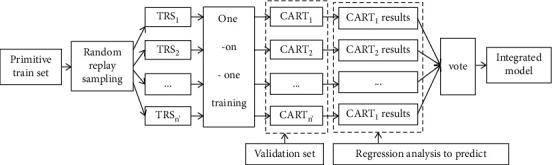
Schematic diagram of random forest modeling principle.

The modeling idea is as follows (see Figure 2): (1) data are randomly selected. Given the original training set *S*_*n*′_={*X*_*n*′_, *Y*_*n*′_}, *X*_*n*′_ ∈ *X* ∈ *R*^*i*^, *n*′=(1.2,…, *n*′), *n*′ is the training set sample size, *n*′ times are randomly selected with replacement, 1 sample is drawn each time, and the probability of the sample being selected is 1/*n*′. This self-help sampling integration method is used to generate *n*′ different train subsets, which are recorded as TRS_1_ ∼ TRS_*n*′_. Samples in different train subsets can be repeated, and samples in the same train subset can also be repeated. Different train subsets are used to train a decision tree model one by one, which is recorded as CART_1_ ∼ CART_*n*′_, and the models are irrelevant. The samples not selected form a validation set to test the generalization ability of CART_1_ ∼ CART_*n*′_. In this paper, the sample size of the original training set *n*′  = 20. (2) Characteristic variables are randomly selected. Each sample has *F* characteristic variables, randomly select *f* characteristic variables from *F* characteristic variables, Specify *f* < *F*, so that the optimal segmentation node can be found at each node to split the decision tree. The whole process does not prune. Then, calculate the best splitting method according to the *f* characteristics, so as to improve the classification performance of CART_1_∼CART_*n*′_. In this paper, *F* = 68. (3) The model is integrated through voting. By using the regression analysis method, the prediction results of CART_1_∼CART_*n′*_ were obtained, and the optimal RF model is integrated by voting.

## 5. Comparative Analysis of Model Operation

### 5.1. Sample Application

Effective sample data numbered 1–39 as *S*={*X*_*n*_, *Y*_*n*_}, *n*=1,…, 39 processed in “2 Sample Data Sources and Preprocessing” in this paper were imported into “*RStudio*” software for model running. Check the training set and testing set established by the software according to the method of “3 Model Index Data Application” in this paper, and the software displays the randomly selected results of the machine. A total of 20 samples (3, 4, 6, 8, 9, 10, 15, 17, 20, 21, 23, 25, 26, 27, 28, 30, 31, 35, 36, 37, 38) were used to construct the original training set. The *K*-fold cross validation method is used to train and optimize the best SVM, BP, Multinom, and RF models, respectively. Another nineteen samples numbered 1, 2, 5, 7, 11, 12, 13, 14, 16, 18, 19, 22, 24, 29, 30, 32, 33, 34, and 39 were used to construct the testing set for the final effect evaluation of SVM, BP, Multinom, and RF models.

### 5.2. Model Fitting Results

**Figure 3:**
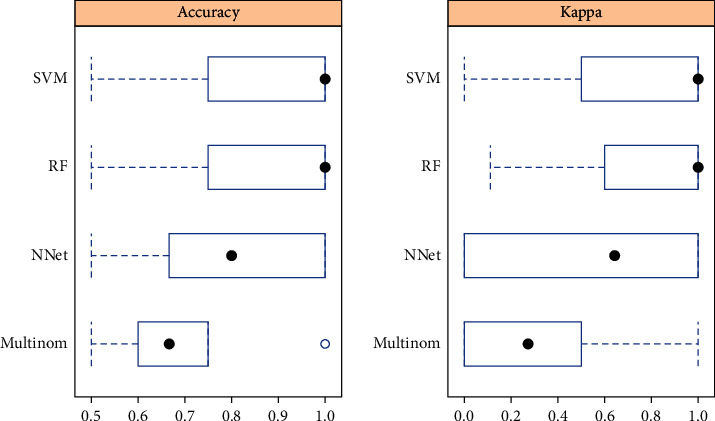
Comparison of accuracy and Kappa median.

Model fitting degree is an index to evaluate the consistency and precision of a theoretical model and actual data. The better the fitting degree is, the higher the consistency and precision will be. The performance of the observation model on the training set is as follows: (1) main parameters: in order to train various optimal machine learning models, it is necessary to continuously improve the main parameters of various model functions to tune the model. “*Rstudio*” software uses the maximum accuracy method to train the four optimal models of SVM, BP, Multinom, and RF, respectively. The running results show that the main parameters of the four models selected in this paper are as follows: SVM kernel function parameter *σ*  = 0.006142, penalty factor *C* = 5; the number of samples selected for one training of BP is size = 5, and the attenuation factor of gradient descent method is decay = 0.1. Multinom determines regression coefficients on a *X*_*n*′_ basis; RF is determined to randomly select *f* = 35 variables from 68 variables and use their optimal segmentation to split the nodes. (2) Comparison of fitting degree: By comparing the accuracy and consistency of the model with Minimum value (as Min), 25% Median (as 1st Qu), Median, Mean, 75% Median (as 3rd Qu.), and maximum (as Max) were compared and found (See Table 3 and Figure 3). RF and SVM fit better.

### 5.3. Prediction Results of the Model

**Figure 4:**
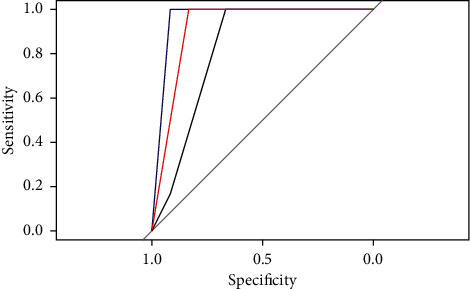
ROC curve of SVM model.

**Figure 5:**
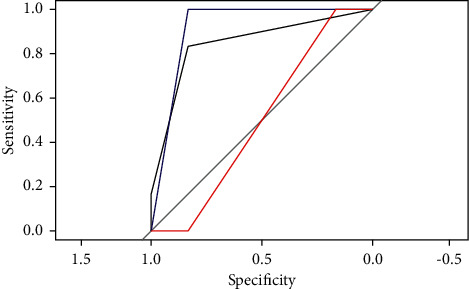
ROC curve of BP model.

**Figure 6:**
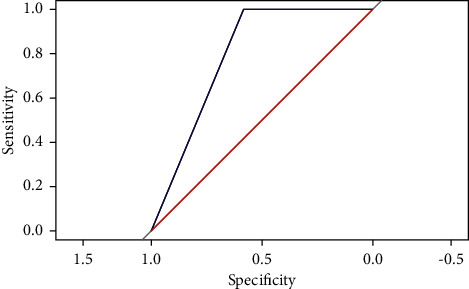
ROC curve of Multinom model.

The SVM, BP, Multinom, and RF models trained on the training set will be used to predict on the testing set. The performance of each model will be observed by the result output by RStudio software. (1) Sensitivity and specificity analysis. These two indexes are used to describe the performance of the classifier. The higher the sensitivity, the lower the leakage rate. The higher the specificity, the lower the misclassification rate. Comparing the sensitivity and specificity of the four models on class excellent, class good, and class medium (see Table 4), we can discover that the RF model has the highest value. (2) Roc curve analysis. The Roc curve is a curve representing subject operating characteristics. The point closest to the upper left corner of the coordinate is the critical value with higher sensitivity and specificity. The larger the area under the curve, the higher the accuracy. By comparing the Roc curves of the four models (see Figures 4–6), it was found that the area under the Roc curve of RF was the largest. (3) Comparison of predicted results. Compare the original project's overall evaluation for each sample in the testing set with the predictions of the four models (see Table 5). The prediction accuracy of the SVM, BP, Multinom, and RF are 73.68%, 68.42%, 73.68%, and 84.21%, respectively, and the RF model has the highest accuracy. Based on the analysis of the model prediction results of the above three aspects, it is concluded that the prediction results of the RF model are the best.

## 6. Conclusion

This paper aims at the whole-process tracking audit problem of “special bonds + PPP” mode (hereinafter referred to as “special bonds + PPP”) in public infrastructure construction projects and establishes an audit evaluation prediction model. Based on theory and methods of machine learning, such as support vector machine, BP neural network, multinomial logistic regression, and random forest, we establish four models, that is, SVM, BP, Multinom, and RF to conduct a research with the following conclusions:

1. Based on the principle of tracking audit coverage in the whole life cycle, take the early project approval decision, medium-term supervision and management, and later handover evaluation as the three dimensions of the whole process tracking audit of “special debt + PPP,” and build an index system containing 68 evaluations, which can express the rationality, economy, efficiency, and sustainable development ability of the project in the whole process.
2. Innovate audit method, apply machine learning artificial intelligence algorithm to audit evaluation prediction, SVM, BP, Multinom, and RF audit evaluation prediction models are established. Through RStudio software, the optimal parameters can be obtained quickly to tune the model and match with the evaluation criteria of the index system. The project survey data stored by the Ministry of Finance are true and effective. The operation of model training and prediction is simple and universal. The results showed that the RF model had a better fitting degree in training, and the sensitivity, specificity, and prediction accuracy of the trained model were the highest among the four models. RF model can greatly improve the efficiency of “special bonds + PPP” audit evaluation and prediction, which has a certain reference significance for carrying out full coverage of audit work.

This paper focuses on how to audit and predict “special debt + PPP” and establishes a matching overall evaluation index system and appropriate audit prediction model. In the future research, we will extend the RF audit prediction model to evaluation prediction in other fields, providing theoretical and intelligent technical support for full coverage of project audit activities of government departments.

## Figures and Tables

**Figure 7:**
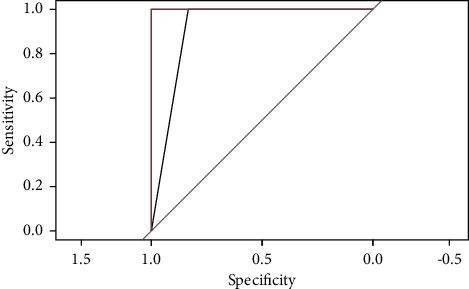
ROC curve of RF model.

**Table 1:** The whole-process tracking audit evaluation index system of “Special bonds + PPP.”

Primary indicators	Secondary indicators	Tertiary indicators
Preliminary project decision stage (A_1_)	Legitimacy of project identification process (B_1_)	Legitimacy of project initiation and screening procedures (C_1_)
		Completeness of procedures and documents (C_2_)
	Rationality of investment estimation decision (B_2_)	Approval process compliance (C_3_)
		Openness and transparency of the evaluation process (C_4_)
		Correctness of preparation basis (C_5_)
		Rationality of estimation accuracy (C_6_)
	Feasibility of preliminary implementation plan (B_3_)	Correctness of scheme preparation basis (C_7_)
		Accuracy of terms and duration of franchise contract (C_8_)
		Rationality of cooperation mode selection (C_9_)
		Legality of commercial contract (C_10_)
		Rationality of investment return mechanism (C_11_)
	Accuracy of value for money reporting (B_4_)	Integrity report of value for money (C_12_)
		Pertinence of qualitative analysis content (C_13_)
		Accuracy of quantitative calculation (C_14_)
		Accuracy of annual discount rate determination (C_15_)
	Demonstration of financial affordability (B_5_)	Integrity of demonstration report (C_16_)
		Accuracy of calculation (C_17_)
		Identification of financial expenditure responsibility (C_18_)
	Rationality of risk sharing (B_6_)	Rationality of risk balance allocation (C_19_)
		Effectiveness of the combination of government resources and social capital (C_20_)
		Whether excessively access to government support (C_21_)
	Compliance of procurement procedures (B_7_)	Completeness of procurement documents (C_22_)
		Transparency of information (C_23_)
		Standardization of response document review (C_24_)
		Compliance of contract signing (C_25_)
	Compliance of special bonds application (B_8_)	Completeness degree of application procedures (C_26_)
		Integrity of budgeting (C_27_)
		Accuracy of bonds accounting (C_28_)
	Solvency of special income (B_9_)	Self-balancing ability of project revenue (C_29_)
		Accuracy of solvency analysis (C_30_)

Mid-term supervision and management stage (A_2_)	The performance of government supervision functions (B_10_)	Adequacy of supervision by relevant departments (C_31_)
		Status of special bonds fund supervision(C_32_)
		Familiarity degree of relevant personnel with special bonds policies and regulations (C_33_)
	Rationality of project financing (B_11_)	Rationality of financing structure (C_34_)
		Economy of financing scheme (C_35_)
		Accuracy of calculation results (C_36_)
	Rationality of special bonds use (B_12_)	Idle rate of special bonds funds (C_37_)
		Connection between special funds and project schedule (C_38_)
		Matching degree of project income period and bonds term (C_39_)
		Transparency of special fund information (C_40_)
	Legality of construction procedures (B_13_)	Rationality of project planning decision (C_41_)
		Completeness of relevant construction procedures (C_42_)
	Performance degree of the project company (B_14_)	Implementation of cooperation agreement (C_43_)
		Management of quality safety and schedule (C_44_)
		Reduction rate of engineering cost (C_45_)
	Mid-term performance assessment quality (B_15_)	Rationality of performance system (C_46_)
		Rationality of the calculation of the incentive effect of the scheme (C_47_)
	Payment status of project completion (B_16_)	Rationality of settlement price (C_48_)
		Rationality of completion settlement preparation (C_49_)
		Rationality of preparation of completion final accounts (C_50_)
	Status of government feasibility gap subsidy (B_17_)	Rationality of stipulated pricing and subsidies (C_51_)
		Correctness of subsidy fund calculation (C_52_)
	Situation of finance and operation (B_18_)	Authenticity and validity of account books and accounting statements (C_53_)
		Transparency of project operation (C_54_)
		Rationality of user charges (C_55_)

Later handover evaluation stage (A_3_)	Standardization of project handover (B_19_)	Compliance of transfer procedures (C_56_)
		Integrity of handover content (C_57_)
		Realization rate of handover standard (C_58_)
	Economy of transfer subsidy amount (B_20_)	Legality of asset appraisal procedures (C_59_)
		Rationality of asset appraisal scheme (C_60_)
	Effectiveness of performance test (B_21_)	Standardization of performance test procedures (C_61_)
		Rationality of performance test scheme (C_62_)
	Repayment degree of principal and interest of special bonds (B_22_)	Repayment rate of principal and interest (C_63_)
		Coverage rate of special income repayment (C_64_)
	Rationality of final performance evaluation (B_23_)	Whether to provide efficient public goods or services (C_65_)
		Public satisfaction (C_66_)
		Environmental impact rate (C_67_)
		Reduction rate of project investment (C_68_)

**Table 2:** Case evaluation sample data processing excerpt.

No	In														
	C_1_	C_2_	C_3_	C_4_	C_5_	C_6_	C_7_	C_8_	…	C_64_	C_63_	C_66_	C_67_	C_68_	OE
1	3	3	3	3	3	3	3	3	…	3	3	3	3	3	Good
2	3	3	3	4	4	3	3	4	…	4	3	3	3	4	Excellent
.	.	.	.	.	.	.	.	.	.	.	.	.	.	.	.
.	.	.	.	.	.	.	.	.	.	.	.	.	.	.	.
.	.	.	.	.	.	.	.	.	.	.	.	.	.	.	.
9	3	3	3	3	2	2	2	2	…	2	3	2	2	2	Medium
10	3	3	3	4	3	2	2	3		4	4	4	4	4	Excellent
11	4	4	4	4	4	3	4	4		4	4	4	4	3	Excellent
12	3	3	3	3	3	3	3	3		2	3	3	3	2	Good
.	.	.	.	.	.	.	.	.	.	.	.	.	.	.	.
.	.	.	.	.	.	.	.	.	.	.	.	.	.	.	.
.	.	.	.	.	.	.	.	.	.	.	.	.	.	.	.
38	4	4	4	4	4	4	4	4	…	4	4	4	4	4	Excellent
39	4	3	4	4	4	4	4	3	…	3	3	4	4	3	Excellent

Note: IN. represents index, No.represents simple number, OE represents overall evaluation of the project.

**Table 3:** Statistical table of model accuracy and Kappa.

Indicators	Model	Min	1st qu	Median	Mean	3rd qu	Max
Accuracy	SVM	0.5	0.7500000	1.0000000	0.8500000	1.00	1
	BP	0.5	0.7500000	1.0000000	0.8333333	1.00	1
	Multinom	0.5	0.6000000	0.6666667	0.7033333	0.75	1
	RF	0.5	0.6666667	0.8000000	0.7933333	1.00	1

Kappa	SVM	0.1111111	0.6	1.0000000	0.7422222	1.0	1
	BP	0.0000000	0.5	1.0000000	0.6666667	1.0	1
	Multinom	0.0000000	0.0	0.2727273	0.3545455	0.5	1
	RF	0.0000000	0.0	0.6428571	0.5285714	1.0	1

Note: Kappa means consistency.

**Table 4:** Statistical table model of sensitivity and specificity.

Indicators	Model	Class: excellent	Class: good	Class: medium
Sensitivity	SVM	0.6667	0.8333	1.00000
	BP	0.7500	0.5000	0.00000
	Multinom	0.5833	1.0000	0.00000
	RF	0.8333	1.0000	1.00000

Specificity	SVM	1.0000	0.7692	0.88889
	BP	0.7143	0.6923	0.94444
	Multinom	1.0000	0.5385	1.00000
	RF	1.0000	0.8462	1.00000

**Table 5:** Comparison of models “RStudio” results and project OE raw data

PO	NO																		
	1	2	5	7	11	12	13	14	16	18	19	22	24	29	30	32	33	34	39
OE	G	E	E	E	G	E	E	M	E	G	E	E	G	E	G	E	E	G	E
SVM	G	E	E	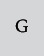	G	E	E	M	E	G	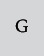	E	G	E	G	E	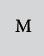	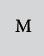	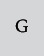
BP	G	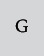	E	E	G	E	E	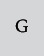	G	G	E	E	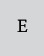	E	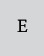	E	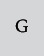	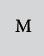	E
Multinom	G	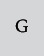	E	E	G	E	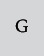	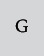	E	G	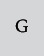	E	G	E	G	E	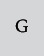	G	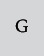
RF	G	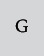	E	E	G	E	E	M	E	G	E	E	G	E	G	E	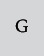	G	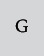

## Data Availability

The data used to support the study are included in the paper.
